# Antibody responses to an inactivated SARS-CoV-2 vaccine in individuals aged from 50 to 102 years

**DOI:** 10.3389/fimmu.2023.1212988

**Published:** 2023-07-31

**Authors:** Hong-Hong Zhu, Guo-Qing Sun, Ji-Yu Wu, Su-Qing Fan, Ying-Ying Zhu, Zhi-Cheng Wang, Xiao-Fang Liao

**Affiliations:** ^1^ Center for Medical Research, Zhejiang Chinese Medical University Affiliated Four-Province-Bordering Hospital of Traditional Chinese Medicine (TCM) – Quzhou Hospital of TCM, Quzhou, Zhejiang, China; ^2^ Department of Medical Laboratory Science, Zhejiang Chinese Medical University Affiliated Four-Province-Bordering Hospital of Traditional Chinese Medicine (TCM) – Quzhou Hospital of TCM, Quzhou, Zhejiang, China; ^3^ Department of Informatics, Zhejiang Chinese Medical University Affiliated Four-Province-Bordering Hospital of Traditional Chinese Medicine (TCM) – Quzhou Hospital of TCM, Quzhou, Zhejiang, China; ^4^ Department of Quality Assurance and Control, Zhejiang Chinese Medical University Affiliated Four-Province-Bordering Hospital of Traditional Chinese Medicine (TCM) – Quzhou Hospital of TCM, Quzhou, Zhejiang, China; ^5^ President for the Zhejiang Chinese Medical University Affiliated Four-Province-Bordering Hospital of Traditional Chinese Medicine (TCM) – Quzhou Hospital of TCM, Quzhou, Zhejiang, China

**Keywords:** inactivated SARS-CoV-2 vaccine, antibody, seroepidemiology, phase 4 trial, chronic disease, hypertension, diabetes

## Abstract

**Objectives:**

To assess antibody responses to an inactivated SARS-CoV-2 vaccine in individuals aged 50 and older.

**Methods:**

We conducted a post-market cross-sectional seroepidemiology study. We recruited 4,632 vaccinated individuals aged 50 and older, measured their total serum SARS-CoV-2-specific antibody (TA), and collected correlates. The primary outcome was the geometric mean titer (GMT) of TA, and the secondary outcome was the decline of TA with age. Univariate, bivariate, and multivariate analyses were used to examine the associations of the TA GMT with age, and trend analyses were used to test whether their associations were significant.

**Results:**

All participants had a detectable TA, which was generally at a low level across all age groups. The TA GMT (95% CI) in AU/mL was 3.05 (2.93, 3.18); the corresponding arithmetic mean (95% CI) was 17.77 (16.13, 19.42) in all participants and 4.33 (3.88, 4.84), 3.86 (3.49, 4.28), 3.24 (2.92, 3.59), 2.77 (2.60, 2.96), and 2.65 (2.48, 2.83) in the age groups of 50-54, 55-59, 60-64, 65-74, and 75 years or older, respectively. The TA GMT decreased with age with a *P*_trend_ < 0.001. The TA GMT was significantly lower in those with hypertension or diabetes compared to those with neither.

**Conclusion:**

The inactivated SARS-CoV-2 vaccine is effective in individuals aged 50 and older. This is the first study that has found an inverse dose-response relationship between ages and the low-level TAs. Older people, especially those with chronic diseases, should get the SARS-CoV-2 vaccine, and their vaccination frequency, dose, and method may need to be different from those of younger people.

## Introduction

The coronavirus disease 2019 (COVID-19), caused by the severe acute respiratory syndrome-coronavirus-2 (SARS-CoV-2), has been pandemic for the past three years, causing incalculable damage to the health and economy of the world. According to data from the Johns Hopkins Coronavirus Resource Center (1), more than 657 million cases and nearly 6.7 million deaths of COVID-19 were confirmed worldwide as of December 2022, with actual incident cases and deaths far more numerous than confirmed ones (2).

SARS-CoV-2 causes more severe cases and deaths in the elderly (3–6). The vaccine helps mitigate the consequences of COVID-19, such as reducing severe cases and deaths among the elderly (3–15). As of 22/12/2022, more than 13 billion doses of the COVID-19 vaccine have been administered worldwide, with more than 3.65 billion doses in China, where 92.61% of people have received at least one dose (1). The first vaccine in China was an inactivated SARS-CoV-2 vaccine, which was approved on 30/12/2020, and found to be safe with acceptable efficacy according to the results of its clinical trials (3–8). Therefore, mass vaccination campaigns have been conducted in China and worldwide. It is necessary and important to conduct post-market epidemiology studies to evaluate its effectiveness in a real-world population.

Among the less than 7% of the population in China who have not received the COVID-19 vaccine (1), a significant portion consists of older people who have been hesitating to get vaccinated because of contraindications and side effects and their anxieties from associated uncertainties. In theory, because SARS-CoV-2 is a brand-new virus to humans, all people, regardless of their age, gender, or disease status, should get the COVID-19 vaccine to acquire SARS-CoV-2-specific antibodies that can provide protection. This is especially important for older people, just like using a small dose of an allergen to treat allergies clinically.

To investigate whether the elderly should get the COVID-19 vaccine, a post-market seroepidemiology study can provide valuable evidence. Therefore, we have designed this study to test our hypothesis regarding the antibody responses of older adults to the inactivated SARS-CoV-2 vaccine.

## Methods

### Study population and design

We conducted a post-market seroepidemiology study, i.e., a phase 4 trial, in the real-world population aged 50 and older after an inactivated SARS-CoV-2 vaccine was approved and a nationwide mass vaccination campaign started in China. This study was approved (approval number: 2022-02-030) by the Ethics Committee of the Quzhou Hospital of Traditional Chinese Medicine (TCM)—Zhejiang Chinese Medical University Affiliated Four-Province-Bordering Hospital of TCM, which is a top-ranking (Third Grade Class A) TCM hospital and is also affiliated with the Red Cross Society of China in Quzhou City.

**Figure 1:**
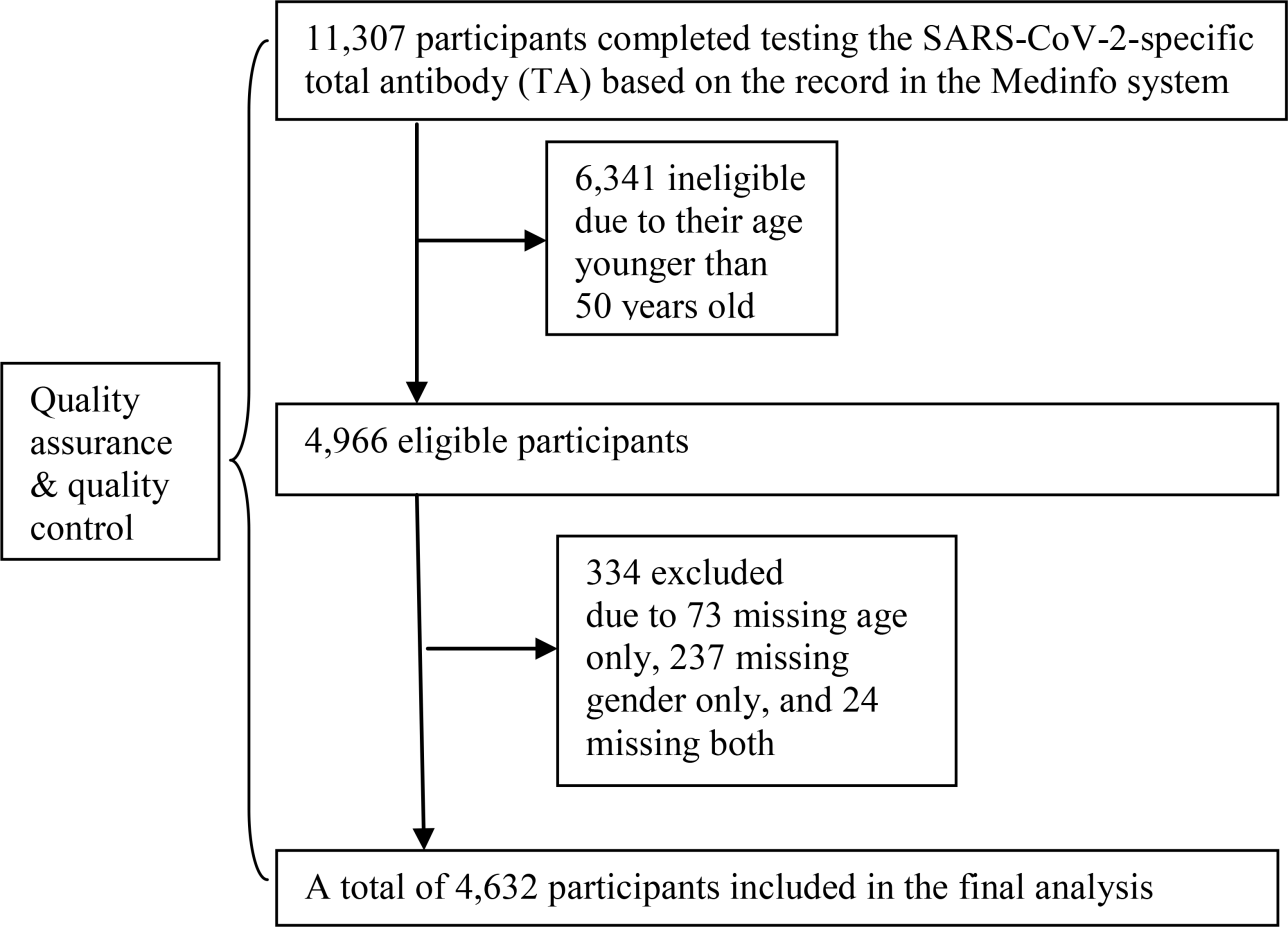
Flow chart of study participant recruitment.

Eligible participants consisted of the elderly who had received at least one dose of the inactivated SARS-CoV-2 vaccine during the period from January to November 2021. Inclusion criteria were as follows: 1) individuals who had completed testing for serum SARS-CoV-2-specific total antibody (TA) at the Quzhou Hospital of TCM; and 2) individuals who aged 50 years or older. Exclusion criteria were as follows: 1) individuals who had tested positive for SARS-CoV-2 RNA before undergoing TA testing; and 2) individuals with missing information. The recruitment of the study participants is shown in **Figure 1**. A total of 11,307 participants completed testing for TA in the hospital from January to November 2021, based on the record in the Medinfo system. A total of 6,341 subjects were ineligible due to their age younger than 50. Finally, 4,632 participants were included in the final analysis after excluding 334 people with missing ages and/or gender.

### Vaccine

The vaccine studied was an inactivated SARS-CoV-2 vaccine produced in Vero cells named COVILO 6·5AU/0·5mL manufactured at the Beijing Institute of Biological Products Co., Ltd., in Beijing, P.R. China (Beijing BIO, CNBG). This vaccine was produced from the 19nCoV-CDC-Tan-HB02 variant.

### Outcomes and covariates

The primary outcome was the serum SARS-CoV-2-specific TA in AU/mL, and the secondary outcome was the inverse association of the TA with age. Covariates included age, gender, and disease status, including hypertension and diabetes.

### Assessment of SARS-CoV-2-specific serum TA titers

A chemiluminescence magnetic microparticle immunoassay was used to measure the serum TA titers in AU/mL. Commercial test kits for the TA (registration no. in China: 20203400198 by Xiamen Wantai Biotech Limited Company, Beijing, P.R. China) were used. Samples were put into a fully automated chemiluminescence immunoassay system, and the test results of an averaged TA titer in AU/mL for each sample were automatically generated with a report from a connected computer.

### Quality assurance and quality control

We conducted quality assurance and quality control before, during, and after the study. The blood samples for the TA were collected by different technicians who were different from those who performed the experiments. The results of the TA were measured by the chemiluminescence magnetic microparticle immunoassay and confirmed by the immune colloidal gold method. We made phone calls to those individuals whose TA was confirmed positive by the immune colloidal gold method to double-confirm that they had received the vaccine, had undergone double-negative SARS-CoV-2 RNA tests, and had no history of SARS-CoV-2 infection. All participants were from an area that never reported COVID-19 cases, as determined by frequent SARS-CoV-2 RNA tests. The authors who executed the TA testing were blinded to the one who extracted the data from the MedInfo system. The corresponding author who designed the study hired two different students who did not know each other to verify the data independently. Finally the corresponding author used STATA software to compare the two datasets with the original one, and finalized the dataset for analyses.

### Statistical analysis

Statistical analysis was conducted with Stata 15 SE software. Age was summarized as a mean and standard deviation (SD). Gender, hypertension, and diabetes status were summarized using counts and percentages. The Pearson *P* value was estimated for age, hypertension, and diabetes status by gender. The geometric mean titer (GMT) and 95% confidence interval (CI) were estimated for the TA, and an individual t-test was used to compare GMT and 95% CI between the two genders. In the final analyses, all variables were defined as follows: age group defined as 50-54 = 0, 55-59 = 1, 60-64 = 2, 65-74 = 3, and 75 years or older = 4; gender defined as woman = 0 and man = 1; hypertension status defined as nonhypertension = 0 and hypertension = 1; diabetes status defined as nondiabetes = 0 and diabetes = 1; and/or hypertension and diabetes status defined as neither hypertension nor diabetes = 0 and either hypertension or diabetes = 1. Univariate, bivariate, and multivariate analyses, including regression and stratification methods, were conducted for the outcome variables. The trend of TA GMT decreasing with age was analyzed, and the *P* value for the trend was estimated for all and each subgroup. All statistical tests were two-tailed. A *P* value ≤ 0.05 was defined as statistically significant; 0.05 < *P* value ≤ 0.10 was defined as statistically marginally significant, and a *P* value > 0.10 was defined as statistically insignificant.

## Results

### Demographic and clinical characteristics

A total of 4,632 eligible participants aged 50 to 102 were included in the final analyses. The characteristics of the study participants overall and by gender are listed in **Table 1**. The mean age (SD) in years was 63.92 (10.21) in 2,512 women and 65.46 (11.41) in 2,120 men; men were on average 1.54 years older than women (*P* = 0.001). Men had a higher prevalence of hypertension (13.07%) than women (10.71%) (*P* = 0.013). Overall, there were no significant differences in diabetes prevalence or SARS-CoV-2-specific TA titers in AU/mL between the two genders.

**Table 1:** Characteristics among vaccinated participants aged 50 years or older in a seroepidemiology study.

Characteristic	Total (N = 4,632)	Women (n = 2,512)	Men (n = 2,120)	*P* value^*^
**Age**, mean ± SD^†^ (years)	64.63 ± 10.81	63.92 ± 10.21	65.46 ± 11.41	0.001
**Hypertension**, n (%)				0.013
Yes	546 (13.36)	269 (10.71)	277 (13.07)	
No	4,086 (88.21)	2,243 (89.29)	1,843 (86.93)	
**Diabetes**, n (%)				0.133
Yes	336 (7.25)	169 (6.73)	167 (7.88)	
No	4,296 (92.75)	2,343 (93.27)	1,953 (92.12)	
**Total antibodies**, GMT (95% CI)^‡^ (AU/mL)	3.05 (2.93, 3.18)	3.04 (2.88, 3.21)	3.07 (2.89, 3.25)	0.980
**Total antibodies**, mean (95% CI) (AU/mL)	17.77 (16.13, 19.42)	18.19 (15.90, 20.48)	17.28 (14.91, 19.65)	0.589

^*^Pearson *P* value, except for the one using an individual test for GMT (95% CI) of the SARS-CoV-2 specific total antibody in AU/mL between women and men.

†SD, standard deviation.

‡GMT (95% CI), geometric mean titer (95% confidence interval).

### TA titer in all 

The GMT (95% CI) of the TA in AU/mL was 3.05 (2.93, 3.18), and the corresponding arithmetic mean (95% CI) was 17.77 (16.13, 19.42) among all 4,632 study participants (**Table 1**).

### TA between women and men

Overall, there was no significant difference in the TA between the two genders (*P* = 0.980) (**Table 1**). The TA GMT (95% CI) in AU/mL was 3.04 (2.88, 3.22) (its corresponding arithmetic mean (95% CI): 18.19 (15.90, 20.48)) in 2,512 women and 3.07 (2.89, 3.25) (its corresponding arithmetic mean (95% CI): 17.28 (14.91, 19.65)) in 2,120 men.

### TA by age

In the stratification analysis (**Table 2**), the TA GMT (95% CI) was 4.33 (3.88, 4.84), 3.86 (3.49, 4.28), 3.24 (2.92, 3.59), 2.77 (2.60, 2.96), and 2.65 (2.48, 2.83) in the age groups of 50-54, 55-59, 60-64, 65-74, and 75 years or older, respectively.

**Table 2:** Univariate analysis: geometric mean titer (GMT) and 95% confidence interval (CI) of the SARS-CoV-2-specific total antibody in AU/mL stratified by age, gender, hypertension, and diabetes status.

Total antibodies, GMT (95% CI), n	Total	Women	Men	Hypertension	Non-hypertension	Diabetes	Non-diabetes
Age group in years							
50 - 54	3.70 (3.31, 4.14), 943	3.75 (3.23, 4.36), 523	3.64 (3.07, 4.31), 420	3.44 (2.88, 5.18), 100	3.73 (3.33, 4.19), 843	3.39 (2.01, 5.70), 61	3.73 (3.32, 4.18), 882
55 - 59	3.49 (3.14, 3.87), 907	3.65 (3.15, 4.22), 517	3.28 (2.83, 3.81), 390	3.21 (2.39, 4.33), 96	3.52 (3.15, 3.94), 811	2.78 (2.06, 3.74), 70	3.55 (3.18, 3.97), 837
60 - 64	3.07 (2.77, 3.40), 721	2.83 (2.48, 3.22), 395	3.39 (2.88, 3.99), 326	3.20 (2.44, 4.19), 121	3.04 (2.73, 3.40), 600	3.17 (2.25, 4.49), 60	3.06 (2.75, 3.41), 661
65 - 74	2.71 (2.54, 2.89), 1,198	2.72 (2.50, 2.96), 668	2.69 (2.44, 2.96), 530	3.15 (2.47, 4.02), 109	2.67 (2.50, 2.85), 1,089	2.65 (2.15, 3.25), 85	2.71 (2.54, 2.90), 1,113
> 74	2.53 (2.37, 2.71), 863	2.38 (2.18, 2.61), 409	2.68 (2.43, 2.95), 454	2.47 (2.06, 2.97), 120	2.54 (2.36, 2.73), 743	2.02 (1.55, 2.64), 60	2.58 (2.40, 2.76), 803
P for trend	< 0.001	< 0.001	< 0.001	0.006	< 0.001	0.003	< 0.001

In the trend analysis, there was a significant inverse association between the TA and age, with a *P* for trend < 0.001. TA significantly decreased with age, whether age was treated as a continuous variable (*P* for trend < 0.001), an age group in 5 years (50-54, 55-59, 60-64, 65-69, 70-74, 75-79, 80-84, and 85 or older) (*P* for trend < 0.001), or an age group defined as 50-54, 55-59, 60-64, 65-74, and 75 or older (some age groups were combined into one group due to similar antibody titers) (*P* for trend < 0.001).

### Univariate analysis for the inverse association of the TA with age

When stratifying the data by gender (**Table 2**), the TA GMT (95% CI) was 3.75 (3.23, 4.36), 3.65 (3.15, 4.22), 2.83 (2.48, 3.22), 2.72 (2.50, 2.96), and 2.38 (2.18, 2.61) in women, and 3.64 (3.07, 4.31), 3.28 (2.83, 3.81), 3.39 (2.88, 3.99), 2.69 (2.44, 2.96), and 2.68 (2.43, 2.95) in men in the age groups of 50-54, 55-59, 60-64, 65-74, and 75 years or older, respectively. In the trend analysis, the inverse association of the TA with age remained significant in women (*P* for trend < 0.001) and in men (*P* for trend < 0.001), respectively.

When stratifying the data by hypertension status (**Table 2**), the TA GMT (95% CI) was 3.44 (2.88, 5.18), 3.21 (2.39, 4.33), 3.20 (2.44, 4.19), 3.15 (2.47, 4.02), and 2.47 (2.06, 2.97) in participants with hypertension, and 3.73 (3.33, 4.19), 3.52 (3.15, 3.94), 3.04 (2.73, 3.40), 2.67 (2.50, 2.85), and 2.54 (2.36, 2.73) in participants without hypertension in the age groups of 50-54, 55-59, 60-64, 65-74, and 75 years or older, respectively. In the trend analysis, the inverse association of the TA with age remained significant in participants with hypertension (*P* for trend = 0.006) and without hypertension (*P* for trend < 0.001), respectively.

When stratifying the data by diabetes status (**Table 2**), the TA GMT (95% CI) was 3.39 (2.01, 5.70), 2.78 (2.06, 3.74), 3.17 (2.25, 4.49), 2.65 (2.15, 3.25), and 2.02 (1.55, 2.64) in participants with diabetes, and 3.73 (3.32, 4.18), 3.55 (3.18, 3.97), 3.06 (2.75, 3.41), 2.71 (2.54, 2.90), and 2.58 (2.40, 2.76) in participants without diabetes in the age groups of 50-54, 55-59, 60-64, 65-74, and 75 years or older, respectively. In the trend analysis, the inverse association of the TA with age remained significant in participants with diabetes (*P* for trend = 0.003) and without diabetes (*P* for trend < 0.001), respectively.

### Bivariate analysis for the association of the TA with age

The TA GMT (95% CI) for each stratum is shown in **Table 3** after stratification by gender and hypertension status. In the trend analysis, the inverse association of the TA with age remained significant in each subgroup of women without hypertension (*P* for trend < 0.001) and with hypertension (*P* for trend < 0.001), and men without hypertension (*P* for trend < 0.001) and with hypertension (*P* for trend < 0.001), respectively.

Table 3Bivariate analysis: geometric mean titer (GMT) and 95% confidence interval (CI) of the SARS-CoV-2-specific total antibody in AU/mL stratified by age and any two factors of gender, hypertension, and diabetes status.

**Table:** 

Total antibodies, GMT (95% CI), n	Women		Men	
	Non-hypertension	Hypertension	Non-hypertension	Hypertension
Age group in years				
50 - 54	3.72 (3.19, 4.35), 469	4.03 (2.29, 7.07), 54	3.75 (3.15, 4.47), 374	2.86 (1.55, 5.28), 46
55 - 59	3.74 (3.20, 4.37), 470	2.83 (1.86, 4.30), 47	3.24 (2.76, 3.79), 341	3.64 (2.35, 5.61), 49
60 - 64	2.82 (2.45, 3.24), 343	2.89 (1.95, 4.29), 52	3.37 (2.82, 4.04), 257	3.44 (2.36, 5.04), 69
65 - 74	2.66 (2.44, 2.89), 608	3.52 (2.46, 5.03), 60	2.68 (2.43, 2.97), 481	2.75 (1.98, 3.82), 49
> 74	2.40 (2.17, 2.65), 353	2.29 (1.86, 2.82), 56	2.68 (2.42, 2.98), 390	2.64 (1.97, 3.54), 64
P for trend	< 0.001	< 0.001	< 0.001	< 0.001

**Table:** 

Total antibodies, GMT (95% CI), n	Women		Men	
	Non-diabetes	Diabetes	Non-diabetes	Diabetes
Age group in years				
50 - 54	3.83 (3.29, 4.47), 500	2.34 (1.04, 5.28), 23	3.59 (3.02, 4.27), 382	4.23 (2.11, 8.50), 38
55 - 59	3.74 (3.20, 4.36), 481	2.65 (1.77, 3.95), 36	3.32 (2.84, 3.88), 356	2.92 (1.83, 4.66), 34
60 - 64	2.79 (2.43, 3.19), 367	3.44 (2.03, 5.82), 28	3.44 (2.90, 4.09), 294	2.96 (1.83, 4.80), 32
65 - 74	2.72 (2.49, 2.98), 620	2.74 (2.08, 3.60), 48	2.70 (2.44, 2.99), 493	2.53 (1.82, 3.52), 37
> 74	2.41 (2.20, 2.64), 375	2.12 (1.36, 3.31), 34	2.73 (2.47, 3.03), 428	1.89 (1.48, 2.41), 26
P for trend	< 0.001	0.015	< 0.001	< 0.001

**Table:** 

Total antibodies, GMT (95% CI), n	Neither hypertension nor diabetes		Either hypertension or diabetes	
Age group in years				
50 - 54	3.69 (3.28, 4.16), 814		3.76 (2.65, 5.32), 129	
55 - 59	3.56 (3.17, 3.99), 786		3.04 (2.37, 3.91), 121	
60 - 64	3.02 (2.70, 3.38), 586		3.27 (2.53, 4.22), 135	
65 - 74	2.67 (2.49, 2.86), 1,031		2.97 (2.49, 3.54), 167	
> 74	2.58 (2.39, 2.78), 690		2.35 (2.02, 2.75), 173	
P for trend	< 0.001		< 0.001	

The TA GMT (95% CI) for each stratum is shown in **Table 3** after stratification by gender and diabetes status. In the trend analysis, the inverse association of the TA with age remained significant in each subgroup of women without diabetes (*P* for trend < 0.001) and with diabetes (*P* for trend = 0.015), and men without diabetes (*P* for trend < 0.001) and with diabetes (*P* for trend < 0.001), respectively.

The TA GMT (95% CI) for each stratum is shown in **Table 3** after stratification by hypertension and diabetes status. In the trend analysis, the inverse association of the TA with age remained significant in participants with neither hypertension nor diabetes (*P* for trend < 0.001) and with either hypertension or diabetes (*P* for trend < 0.001), respectively.

### Multivariate analysis of the association of TA with age

After adjusting for gender, hypertension, and diabetes status in the multivariate regression model, the inverse association of the TA with age was still significant (*P* for trend < 0.001). The adjusted factors of gender, hypertension, and diabetes status were also shown significant in the final model. 

The TA GMT (95% CI) for each stratum is shown in **Table 4** after stratification by gender, hypertension, and diabetes status. In the trend analysis, the inverse association of the TA with age remained significant in each subgroup of women with neither hypertension nor diabetes (*P* for trend < 0.001), women with either hypertension or diabetes (*P* for trend < 0.001), men with neither hypertension nor diabetes (*P* for trend < 0.001), and men with either hypertension or diabetes (*P* for trend < 0.001).

**Table 4:** Multivariate analysis: geometric mean titer (GMT) and 95% confidence interval (CI) of the SARS-CoV-2-specific total antibody in AU/mL stratified by age, gender, hypertension, and diabetes status.

Total antibodies, GMT (95% CI), n	Women		Men	
	Neither hypertension nor diabetes	Either hypertension or diabetes	Neither hypertension nor diabetes	Either hypertension or diabetes
Age group in years				
50 - 54	3.72 (3.18, 4.35), 464	4.03 (2.38, 6.82), 59	3.66 (3.06, 4.39), 350	3.54 (2.19, 5.71), 70
55 - 59	3.78 (3.22, 4.43), 458	2.77 (1.97, 3.88), 59	3.27 (2.78, 3.85), 328	3.34 (2.30, 4.84), 62
60 - 64	2.78 (2.42, 3.19), 336	3.13 (2.13, 4.62), 59	3.39 (2.82, 4.08), 250	3.38 (2.39, 4.79), 76
65 - 74	2.64 (2.42, 2.89), 576	3.31 (2.54, 4.31), 92	2.71 (2.43, 3.01), 455	2.60 (2.06, 3.27), 75
> 74	2.42 (2.19, 2.67), 322	2.25 (1.82, 2.79), 87	2.73 (2.45, 3.05), 368	2.46 (1.96, 3.09), 86
P for trend	< 0.001	< 0.001	< 0.001	< 0.001

## Discussion

This post-market seroepidemiology study, i.e., a phase 4 trial, found that all vaccinated participants had a detectable TA, which was at a low overall level across all age groups and decreased with age, with a significant trend in the real-world population aged 50 to 102. This inverse association was observed consistently across all groups, including each subgroup of women, men, participants with or without hypertension, participants with or without diabetes, women with or without hypertension, men with or without hypertension, women with or without diabetes, men with or without diabetes, women with either hypertension or diabetes, women with neither hypertension nor diabetes, men with either hypertension or diabetes and men with neither hypertension nor diabetes.

Our findings that all participants had a detectable TA, which was overall at a low level across all age groups, can be explained as follows: 1) SARS-CoV-2 is a brand-new virus for this population, so each participant is supposed to have some TA responses to the inactivated vaccine, regardless of their age, gender, and disease status; 2) the low level of TA was probably due to the age range of 50 to 102 years since immunity decreases with age; 3) the low level of TA could be due to the natural degeneration of antibodies in a small number of seniors whose blood samples were taken more than 6 months after vaccination, the variance of the early stage antibody level within 2 weeks after vaccination, or the variance of antibody responses to the vaccine between individuals, which are the real facts that all post-market epidemiological studies must face; and 4) the average TA level in our study is consistent with the reported results of this inactivated vaccine (3–7, 9, 10, 13).

Our finding of a low TA level across all age groups indicates that the vaccination frequency, dose, and method for seniors may need to be different from those for the population younger than 50 years of age to increase antibody levels. However, we believe that this level of TA titers would still provide some protection from COVID-19 to these seniors, which is at least much better than nothing. Also, when people are vaccinated with this inactivated SARS-CoV-2 vaccine, the SARS-CoV-2-specific memory B and T cells are detectable, and a large amount of SARS-CoV-2-specific CD4^+^ and CD8^+^ T cells are produced after the third dose of this vaccine (4, 6, 16), which would provide some additional protection.

Our study was conducted in an area where no actual COVID-19 cases were diagnosed based on the results of frequent SARS-CoV-2 RNA testing. TA titers were measured in 2021 when the dynamic zero-COVID policy was successfully applied. For those whose TA was confirmed positive by the immune colloidal gold method, we made phone calls to confirm that they all had the inactivated vaccine, double-negative SARS-CoV-2 RNA tests, and had never had SARS-CoV-2 infection before. Therefore, the measured TA in our study can be confirmed by the responses to the COVID-19 vaccine instead of the SARS-CoV-2 infection.

This is the first study to find an inverse association of the TA responses to an inactivated vaccine with age in the real aging population, although their overall TA was at a low level across all age groups. It is usually much harder to find a dose-response relationship when the response is in the low-level range. In addition, this inverse dose-response relationship between the TA and age was consistent before and after controlling for the confounders and effect modifiers studied. The inverse association also remained consistent when age was defined differently. Age is intuitively associated with immunity; the older the lower their immunity in general. Therefore, the TA responses to the inactivated vaccine in this elderly population would be expected to be low and should diminish with age because their immunity declines with age. This indicates that the older the people aged 50 or older get, the more frequent vaccination with or without a higher dose vaccine and/or different vaccination methods such as sniff-able, inhalable, or spray vaccine may need to be given.

Our finding of a significant inverse association of TA with age is indirectly supported by a qualitative study (n = 205 participants) of serum IgG testing after booster vaccination with the same inactivated vaccine as in our study (15), which found that a significantly lower IgG positive rate (58.3%) of COVID-19 antibodies in the group aged over 50 years following a booster dose than that (86.0%) in those aged 18 to 50. Our finding is also supported by a study of 57 elderly people who were vaccinated with an inactivated vaccine but became infected with SARS-CoV-2 (7).

Gender was overall not associated with the TA in our study, which is consistent with other studies (3, 7, 14, 15), but in the multivariate analyses, gender was shown to be a significant confounder in the final model in our research, which may be due to the residual confounding of age between women and men or other factors not studied.

SARS-CoV-2 infects humans through angiotensin-converting enzyme 2 (ACE2) receptors on host cells (3, 16). ACE2 is believed to be involved in the development of hypertension and diabetes mellitus (17), which may be related to why elderly patients with chronic diseases have a higher incidence of severe cases and deaths of COVID-19. Our study found that hypertension was significantly associated with TA but not with diabetes, which may be due to the fact that the number of diabetes mellitus cases is smaller than that of hypertension. When hypertension and diabetes mellitus are combined into one variable, the variable is shown to be significant in the final multivariate regression.

Also, we found that the TA was lower in those with hypertension or diabetes than in those with neither hypertension nor diabetes, which may explain why senior cases with chronic diseases have a higher incidence of severe cases and deaths of COVID-19 (3–7, 17). In addition, our finding that the variation in TA is greater in the elderly with hypertension or diabetes than in those without indicates that other factors not examined in our study need to be further investigated. More research needs to be done on the exact biological mechanism by which the elderly with hypertension and/or diabetes are associated with low TA. Vaccination is believed to be the most important way to control the COVID-19 pandemic before curative drugs are invented. Older people with hypertension and diabetes should be encouraged to get this vaccine, but their frequency, dose, and method of vaccination may also need to be different from those administered to populations younger than 50.

This study has several limitations. Our results were based on a cross-sectional dataset of individuals aged 50 to 102 years old, which limits generalization to a general population. Only one serum sample was used to measure TA, which is likely to be the case in many epidemiological studies due to limited budgets for large sample sizes. Our results were only adjusted for some of the correlates studied. They need longitudinal data with a large sample size to be confirmed.

Our findings, on the other hand, are valid because 1) this is a post-market epidemiology study, i.e., a phase 4 trial, whose results are more reliable than those from phase 1 to phase 3 trials; 2) our results were based on real-world subjects aged 50 to 102 who participated in nationwide mass vaccination campaigns, which should provide solid evidence for seniors; 3) the inverse dose-response relationship between the TA and age was found to be significant, although it was overall at a low level across all age groups; 4) the significant inverse dose-response relationship remained consistent when age was defined differently and before and after adjustment for important confounders and/or effect modifiers; 5) our large sample size (n = 4,632) guaranteed the stability of our results; and 6) importantly, our findings from this post-market seroepidemiology study are supported by other works (5–7, 9–10, 13).

In conclusion, this inactivated SARS-CoV-2 vaccine is effective for the elderly, and the vaccination frequency, dose, and method for seniors may need to be different from those for the younger population in order to increase their TA titer. This is the first study that has found a consistently significant inverse dose-response relationship between the TA responses to inactivated vaccine and age, although the TA was at a low level overall across all age groups and was lower in the elderly with hypertension and/or diabetes compared to those with neither in the real-world population aged 50 to 102. We suggest that seniors, especially those with chronic diseases, should be especially encouraged to get the COVID-19 vaccine, considering the fact that humans may be living with SARS-CoV-2 in the long run. Our results need to be confirmed by epidemiologic studies with larger sample sizes and longitudinal data.

## Data availability statement

The raw data supporting the conclusions of this article will be made available by the authors, without undue reservation.

## Ethics statement

This study was approved (Approval NO: 2022-02-030) by the Ethics Committee of Quzhou Hospital of Traditional Chinese Medicine (TCM)—Zhejiang Chinese Medical University Affiliated Four-Province-Bordering Hospital of TCM. The patients/participants provided their written informed consent to participate in this study.

## Author contributions

HHZ, GQS, JYW, and SQF managed the participants and data collection; HHZ conceptualized the work, designed the study, developed the strategic plan, cleaned and analyzed the data, interpreted the data, drafted, revised, and submitted the manuscript; GQS, SQF, and YYZ performed the experiments; HHZ, SQF, ZCW, and XFL did the quality assurance and quality control for this study. All authors contributed to the article and approved the submitted version.
